# Contrast-enhanced ultrasound in renal cystic lesions: an update

**DOI:** 10.1007/s10396-024-01489-x

**Published:** 2024-08-20

**Authors:** Federica Masino, Laura Eusebi, Michele Bertolotto, Sara Maria Pizzileo, Francesco Pizzolorusso, Giuseppe Sortino, Lucia Pitoni, Stefano Santarelli, Andrea Benedetto Galosi, Giuseppe Guglielmi

**Affiliations:** 1 Department of Clinical and Experimental Medicine, Foggia University School of Medicine, Viale L. Pinto 1, 71121 Foggia, Foggia Italy; 2 Radiology Unit, “Carlo Urbani” Hospital, Via Aldo Moro 52, 60035 Jesi, Ancona Italy; 3 Radiology Unit, “Cattinara” Hospital, Trieste University, Strada di Fiume 447, 34149 Trieste, Triestino Italy; 4 Urology Unit, “Carlo Urbani” Hospital, Via Aldo Moro 52, 60035 Jesi, Ancona Italy; 5 Nephrology Unit, “Carlo Urbani” Hospital, Via Aldo Moro 52, 60035 Jesi, Ancona Italy; 6 Urology Unit, “Riuniti Torrette” Hospital di Ancona, Via Conca 71, 60126 Torrette, Ancona Italy; 7 Radiology Unit, “Dimiccoli” Hospital, Viale Ippocrate 15, 70051 Barletta, Barletta-Andria-Trani Italy; 8 Radiology Unit, IRCCS Casa Sollievo della Sofferenza” Hospital, Viale Cappuccini 1, 71013 San Giovanni Rotondo, Foggia Italy

**Keywords:** Contrast-enhanced ultrasound, CEUS, Kidney cyst, Cystic lesion, Diagnostic imaging

## Abstract

This narrative review aims to describe the current status of contrast-enhanced ultrasound (CEUS) in characterizing renal cystic lesion. The imaging techniques usually performed for their evaluation are ultrasonography (US), computed tomography (CT), and magnetic resonance imaging (MRI) with different criteria of application based on the individual case and the purpose of the examination. Generally, US, as a non-ionizing examination, is the first imaging modality performed and therefore the one that incidentally detects cystic lesions. CT is the most performed imaging modality for cystic lesion assessment before MRI evaluation. It provides better characterization and management and has been introduced into the Bosniak classification. In this context, CEUS is making its way for its characteristics and represents the emerging technique in this field. With these premises, the authors analyze the role of CEUS in the evaluation of renal cysts, starting with an explanation of the technique, describe its main advantages and limitations, and end with a discussion of its application in the Bosniak classification and management, following the current major guidelines.

## Introduction

Renal cysts represent the most prevalent kidney lesions encountered as an incidental finding in patients undergoing abdominal imaging [1]. Published data, primarily derived from ultrasonography (US) and computed tomography (CT), suggest a prevalence of 40% in the adult population and 4% in pediatric patients, making it the most common kidney abnormality [1, 2].

Imaging plays a critical role in the evaluation of renal cysts, with US representing the first approach with the highest rate of detection, and with CT scans and magnetic resonance imaging (MRI) as the most performed techniques for characterization. Contrast-enhanced ultrasound (CEUS) might represent a possible emerging modality in this field because of its various advantages, which will be discussed in this narrative review.

Most renal masses are benign cysts and are incidental findings, but there is a small subgroup of a malignant nature. The evaluation of renal cysts has evolved with updates to the Bosniak classification and other guidelines. In general, use of such terms as “complicated cyst” or “complex cyst” should be avoided, even though these terms are common in clinical practice for indeterminate renal cysts that require further characterization. Therefore, complicated or complex renal cysts pose challenges for accurate characterization through US and usually necessitate contrast agent administration [3]. Due to the absence of ionizing radiation and its cost-effectiveness, CEUS is emerging as a valuable alternative to contrast-enhanced CT and MRI.

**Fig. 1:**
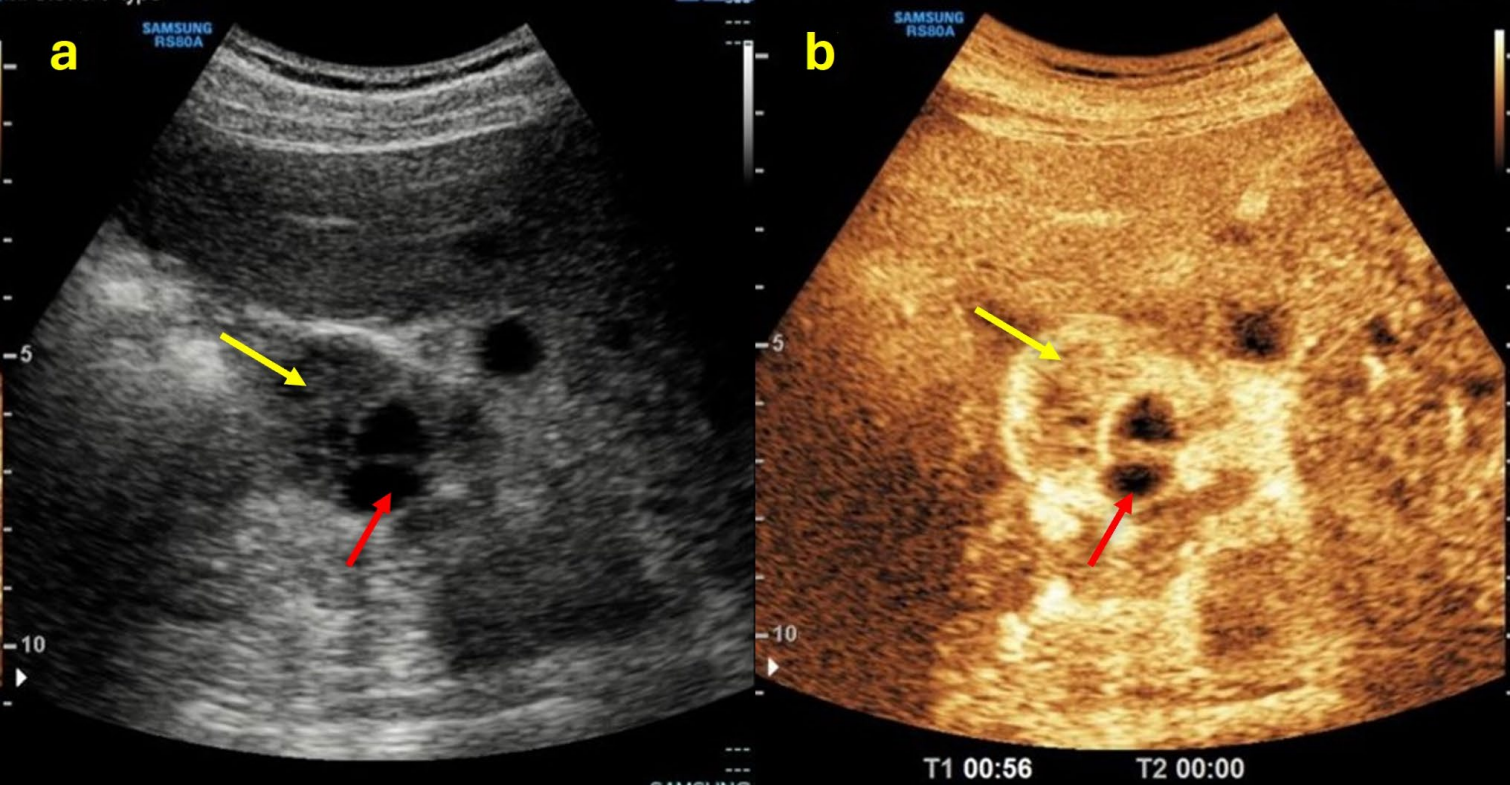
**(a, b)** B-mode US images **a** showed the presence of a renal mass with a mixed echogenic structure made up of both solid (yellow arrow) and cystic (red arrow) components; CEUS image **b** showed a better characterization of the mass presenting more than 25% of enhancement of the solid component. The patient underwent a histological biopsy examination that revealed a Grade 1–2 renal cell carcinoma of the clear cell type with diffuse hemorrhagic foci

Although most renal cysts are benign and pose no threat to the individual, a wide range of potential differential diagnoses and malignancies, such as renal cell carcinoma (RCC), may also present a cystic component (approximately 15% of cases), the so-called “cystic RCC” [4]. Misinterpreting a malignant renal cystic lesion can have severe consequences for the patient [5]. For these reasons, it is critical in the evaluation of a renal mass to determine whether it is cystic or solid in order to ensure proper patient management and to choose the most appropriate type of imaging. The 2019 version of the Bosniak classification system considers 25% to be the enhancement threshold for discriminating solid from cystic lesions. Accordingly, a renal lesion with solid tissue with an enhancement of less than one-quarter should be considered as cystic, and solid if the enhancement is greater than one-quarter [6] (Fig. 1). This article provides a straightforward method for assessing renal cystic lesions that are simple cysts or suspected of malignancy. The radiologist plays a crucial role in this procedure.

## Renal contrast-enhanced ultrasound

### Technique

CEUS is a novel technology that reflects tissue perfusion, using a US contrast agent (UCA). The most commonly used UCA is a second-generation contrast agent, sulfur hexafluoride, which is made of microbubbles and a low-solubility gas enveloped by a phospholipid shell.

UCA, injected intravenously, is a blood pool tracer that never leaves the blood vessel and can be used for real-time dynamic imaging of microcirculation perfusion. UCA remains in the circulation for a period sufficient to reach the organ and guarantee an adequate interpretation of post-contrast graphic phases. To perform a CEUS examination, it is necessary to have a US system equipped with microbubble-specific technology, capable of separating the signal coming from microbubbles (non-linear) from that of stationary tissues (linear) [7]. A B-mode US evaluation should be performed before the contrast-enhanced approach.

After contrast injection, enhancement can be detected in real time for up to 5–7 min in the liver or spleen, while the kidneys enhance for a shorter time, about 2 min. Unlike contrast-enhanced CT (CECT), CEUS allows us to obtain only the cortical phase (15–30 s after UCA administration) and the parenchymal phase (25 s – 2 min after UCA administration), while the excretory phase is missing due to the respiratory elimination pathway of the UCA [8]. The kidneys are remarkably vascularized organs, receiving approximately 21% of cardiac output. The UCA starts filling the arterial pedicle and the main branches: segmental arteries, lobar arteries, and arciform and interlobar arteries. As a result, the cortex is enhanced after a few seconds, followed by the cortico-medullary junction, best visible in a slow-motion video, and lastly by medullary perfusion with the outer medulla filling in earlier and the pyramids filling in gradually later. As time passes, the UCA concentration in the circulation decreases, and enhancement fades. However, in cases of chronic renal insufficiency, the enhancement is less evident and decreases sooner [6, 7]. Any anomalous enhancement pattern compared to the cortex enhancement could be related to malignancy [8].

An experienced radiology specialist with genitourinary US imaging skills performed the US examination reported in this narrative review using a convex probe (3.5–5-5 MHz) on a RS85 Prestige Samsung Ultrasound system. CEUS images were obtained after intravenous injection of 1.4–2.4 mL of a second-generation UCA, followed by 5–10 mL of 0.9% saline solution.

### Advantages and limitations

US is the main imaging modality performed for the detection of incidental renal masses: 83% of them are diagnosed with US and 14.4% with CT scans [10, 11]. The main reason for this is that US is a first-level investigation selected as the first technique in the patient's abdominal evaluation as it does not involve the use of ionizing radiation. However, B-mode US often fails to define whether the lesion is solid or cystic, a fundamental distinction for patient management, since solid lesions are more likely malignant. From this perspective, CEUS should be performed to complete the US examination in order to obtain additional data regarding contrast enhancement.

Like US, CEUS is a widely available method because of its safety and cost-effectiveness, and at the same time CEUS overcomes some of the main issues of US, such as being contrast-free and having low accuracy and specificity. In addition, CEUS can reduce the inter-observer variability that is typical of the US modes, as it allows quantitative evaluation through time/signal intensity curves [12].

UCAs, unlike iodine and gadolinium-based contrast agents used respectively in CT and MRI, do not have an extravascular phase, and consequently do not provide information on capillary permeability and the excretory phase. However, since UCAs are blood pool enhancers and extremely sensitive in detecting microcirculatory anomalies, and thanks to their capability of being confined to the blood vessels, they guarantee the maximum spatial resolution typical of US [12, 13].

CEUS is safer than other imaging modalities in terms of related side effects to contrast agent administration. UCAs are injected in low quantities, in an order of a few millimeters, and are not excreted through the kidneys but rather largely through breathing and a very small amount through the liver. Consequently, a creatinine evaluation is not necessary, and UCAs can be administered to patients with renal insufficiency without risk of causing contrast-related nephropathy or nephrogenic systemic fibrosis. The European Federation of Societies for Ultrasound in Medicine and Biology (EFSUMB) guidelines state that UCAs do have a lower rate of anaphylactoid reactions versus iodinated contrast agents and are comparable to that of gadolinium contrast agents. However, moderate adverse reactions, such as headache, nausea, and chest pain, occur more frequently but at the same time resolve spontaneously in a short time and without repercussions [14].

CEUS is mentioned in the European Association of Urology (EAU) guidelines as having sensitivity (the ability to correctly detect the lesion), specificity (the ability to correctly exclude the lesion), and negative predictive value (the reliability of a negative examination result) close to 100% [7, 15, 16].

Regarding renal pathology, the limitations of CEUS are substantially comparable to those found in US. The artifacts are similar: posterior acoustic barrage due to bowel gas or ribs, beam attenuation in deep lesions, and posterior acoustic barrage in case of calcifications. In the latter case, calcifications do not allow a complete assessment of the lesion, as well as in the case of a deep position of the cyst. In addition, in patients with a high body mass index, US and CEUS are not the most appropriate investigations and might suffer a significant loss of sensitivity and specificity. Very small lesions might be missed, and this represents an important limitation of US and CEUS [16, 17].

Even if CEUS examinations are not technically difficult, the ability to perform the investigation and its repeatability are the most important obstacles in clinical practice. For this reason, the execution of CEUS requires adequate operator training. Only by using an expert operator can CEUS be an effective and repeatable imaging technique [9].

## Contrast-enhanced ultrasound in kidney cystic lesion characterization

### Bosniak classification

To standardize the characterization and management of renal cysts in 1986, Professor Bosniak proposed a radiological classification of renal cysts following CT imaging features. Four classes according to the degree of malignancy risk were proposed. In 1997, class II was split by Bosniak into two separate subclasses (II and IIF, where the F stands for follow-up), leading to five classes in the Bosniak classification [18]. In 2012, Bosniak defined I and II masses as “clearly benign,” IIF masses as “probably benign,” III masses as “indeterminate,” and IV masses as “clearly malignant”. These adaptations were fundamental in the management of lesions and allowed radiologists and urologists to identify specific recommendations [18].

In 2004, Israel et al. proposed an update to the Bosniak classification since their study had shown that CT and MRI findings were similar in most cystic renal masses [19]. In 2019, Silverman et al. proposed an update to the Bosniak classification, resulting in Bosniak classification 2019 (BC-2019), with the following goals: reduce inter-observer variability, increase specificity, establish specific definitions for imaging features, improve the precision rate of malignancy within each category, and reduce masses undergoing unnecessary treatment by placing a greater proportion of lesions in the lower classes. The 2019 version formally incorporates MRI into the classification, includes specific definitions for individual imaging features and Bosniak classes, incorporates a larger proportion of renal masses encountered in clinical practice (e.g., incompletely characterized but highly likely benign cysts), and enables a greater proportion of masses to be placed into lower Bosniak classes. Partly based on evidence and partly on everyday clinical practice, BC-2019 requires further validation before widespread application. The role of US was not clarified in the 2019 classification [18].

In 2020, EFSUMB proposed a further adaptation of the Bosniak classification with the same five classes (I, II, IIF, III, IV) but considering imaging features of renal cysts on CEUS. This classification aimed to increase standardization and reduce inconsistencies and ambiguities. Moreover, it defined situations in which CEUS was not indicated or was superior to CT and MRI.

**Fig. 2:**
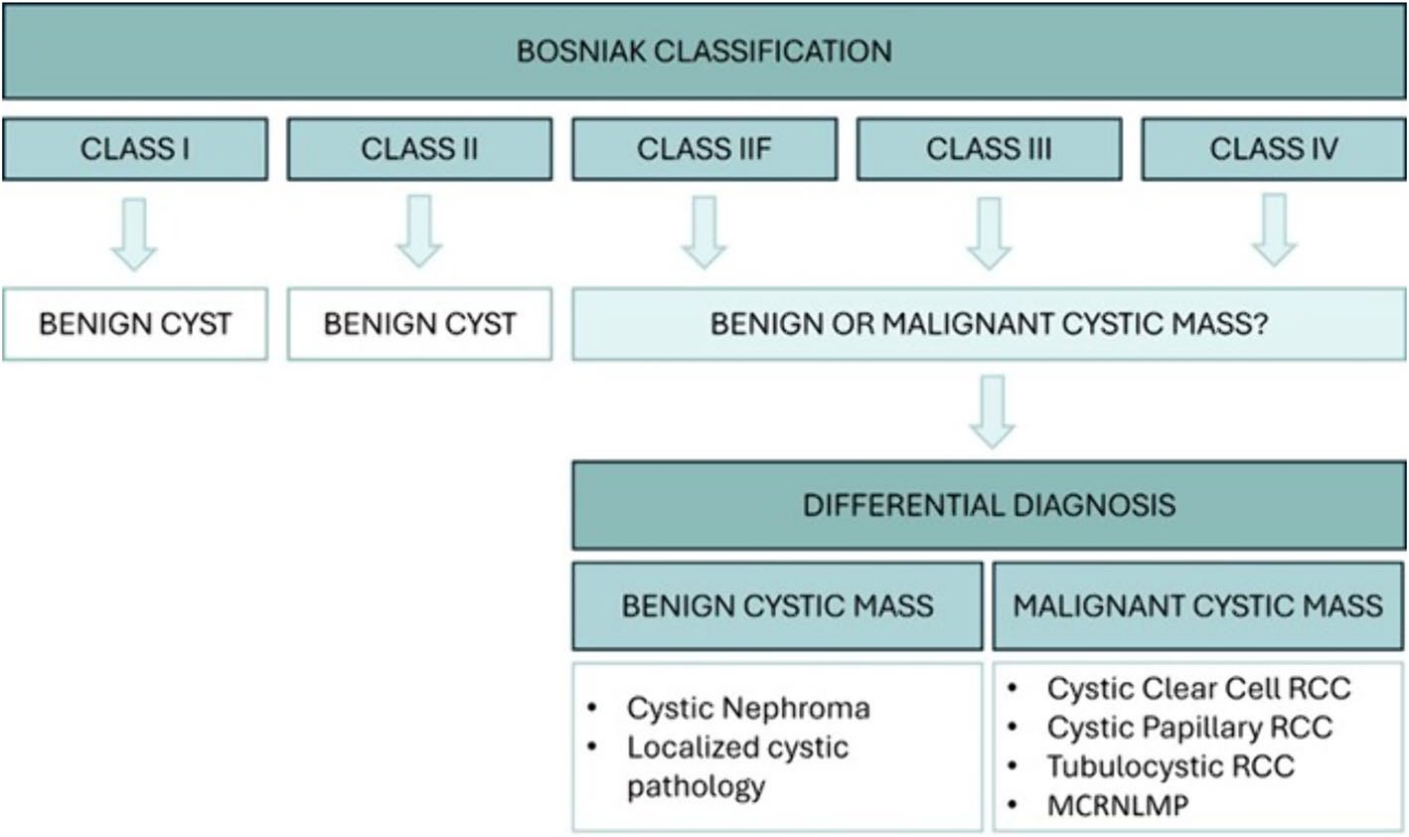
Summary diagram of the Bosniak classification with major differential diagnoses for class IIF to class IV. RCC: renal cell carcinoma; MCRNLMP: multilocular cystic renal neoplasm of low malignant potential

Thus, the Bosniak classification categorizes renal cystic lesions and currently comprises five classes (I, II, IIF, III, IV), with renal lesions from class IIF to IV entering into the differential diagnoses between benign and malignant lesions (Fig. 2). The Bosniak classification should not be applied to masses with an infectious, inflammatory, or vascular etiology that might appear cystic; all other renal cystic masses should be assessed via imaging for complete characterization [6].

In determining the type of class according to Bosniak, it is necessary to start the examination with B-mode US, taking into account the thickness of the wall, the margins, the content, and the presence of septa (structures in a cystic mass that connect two surfaces). Lesion size is not a consideration for cyst categorization with conflicting results for predicting malignancy. Similarly, calcifications are not important in the classification of cystic kidney lesions. What is important to consider and evaluate is the presence of a vascularized tissue component, which takes contrast, within the renal lesion under examination [20].

**Fig. 3:**
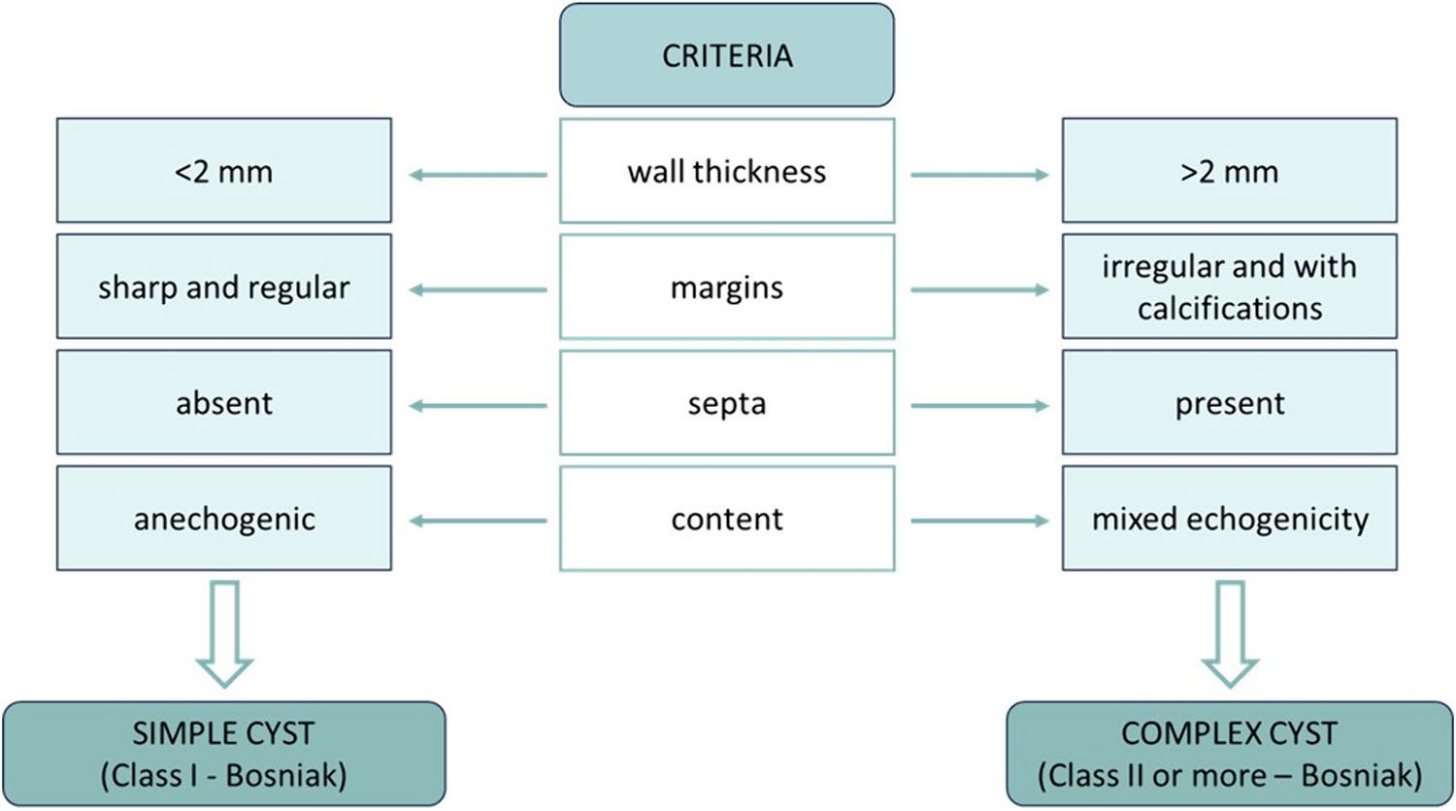
Summary diagram of the criteria for distinguishing simple from complex cysts

As a first approach, the characteristics listed are assessed in B-mode to differentiate simple and complex cysts. A simple cyst is represented by class I according to Bosniak, while a complex cyst belongs to class II or higher according to Bosniak (Fig. 3). Although terms such as “simple” and “complex” are frequently used in clinical practice, they should generally be avoided as they become ambiguous in defining the kind of renal cysts that require additional definition based on CT, MRI, or CEUS [6].

When assessing a cystic lesion, it is useful not to forget the advantages that a US study provides, such as the presence of artifacts of diagnostic value. For example, posterior acoustic enhancement indicates that the content of the kidney lesion is liquid. Depending on the US features, it could be useful to complete the examination with injection of a UCA. While the characterization of simple cysts and a subset of minimally complicated benign cysts is achieved through B-mode US, most complex renal cysts are effectively characterized using CEUS.

**Fig. 4:**
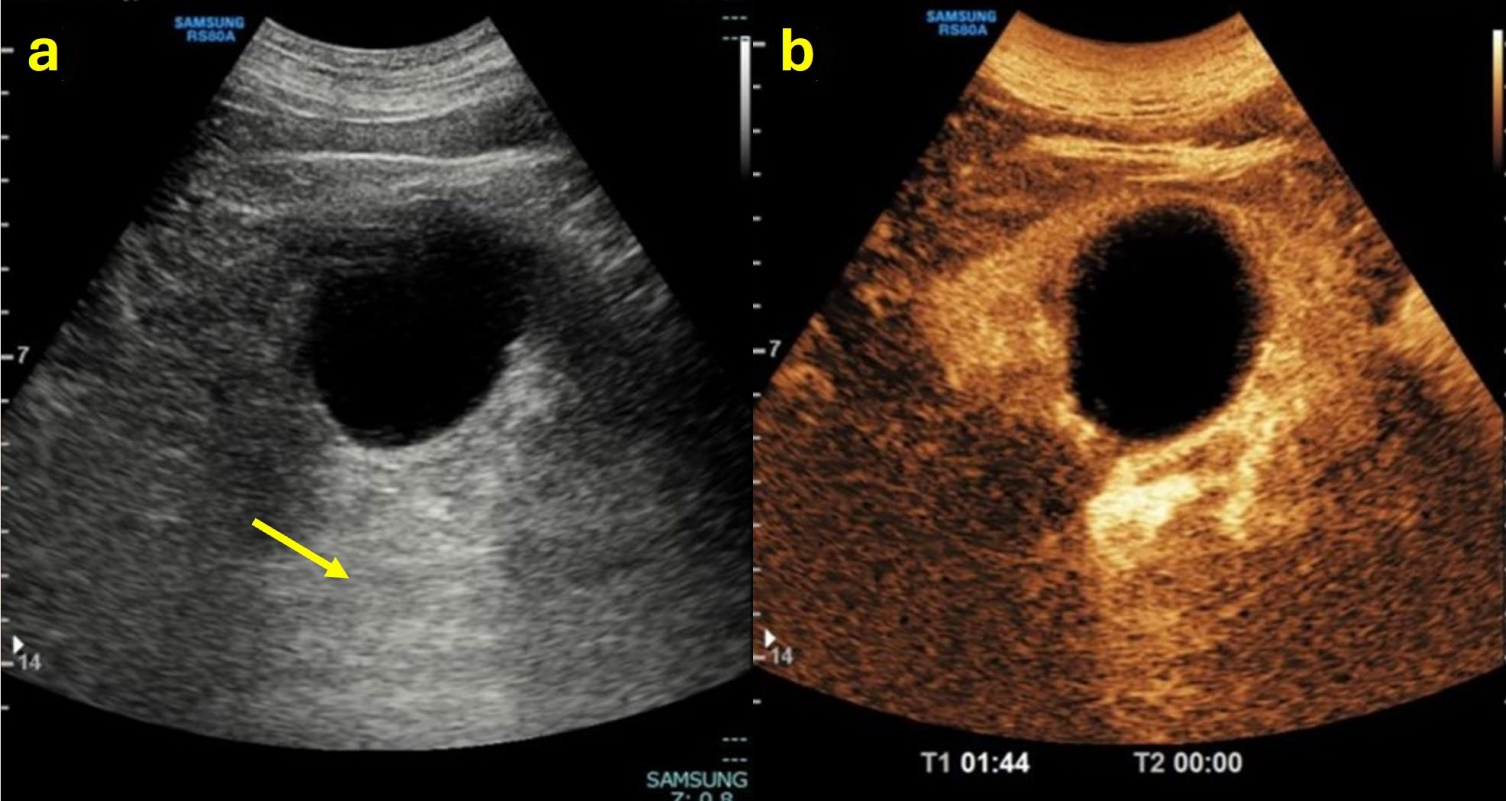
**(a, b)** B-mode US image **a** showed an anechoic cortical lesion with a posterior enhancement artifact (yellow arrow) indicative of cystic content; CEUS image **b** confirmed the cystic nature of the renal lesion since it showed no contrast enhancement. The lesion was classified as Bosniak I

In the case of a cystic lesion that shows the characteristics of a Bosniak class I cyst, completing the examination with CEUS is not necessary. Therefore, B-mode evaluation is sufficient since it fully characterizes this kind of cyst (Table 1). If CEUS is performed anyway, it would show a thin wall without irregularities with no enhancement or individual microbubbles running within tiny vessels in the wall (Fig. 4).

**Table 1:**
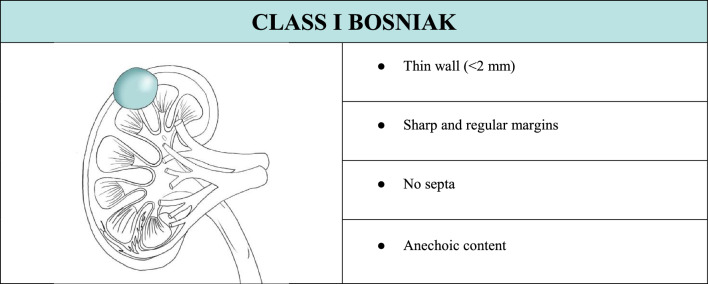
Summary of ultrasound features to define a class I Bosniak cyst, associated with explanatory iconographic image

**Fig. 5:**
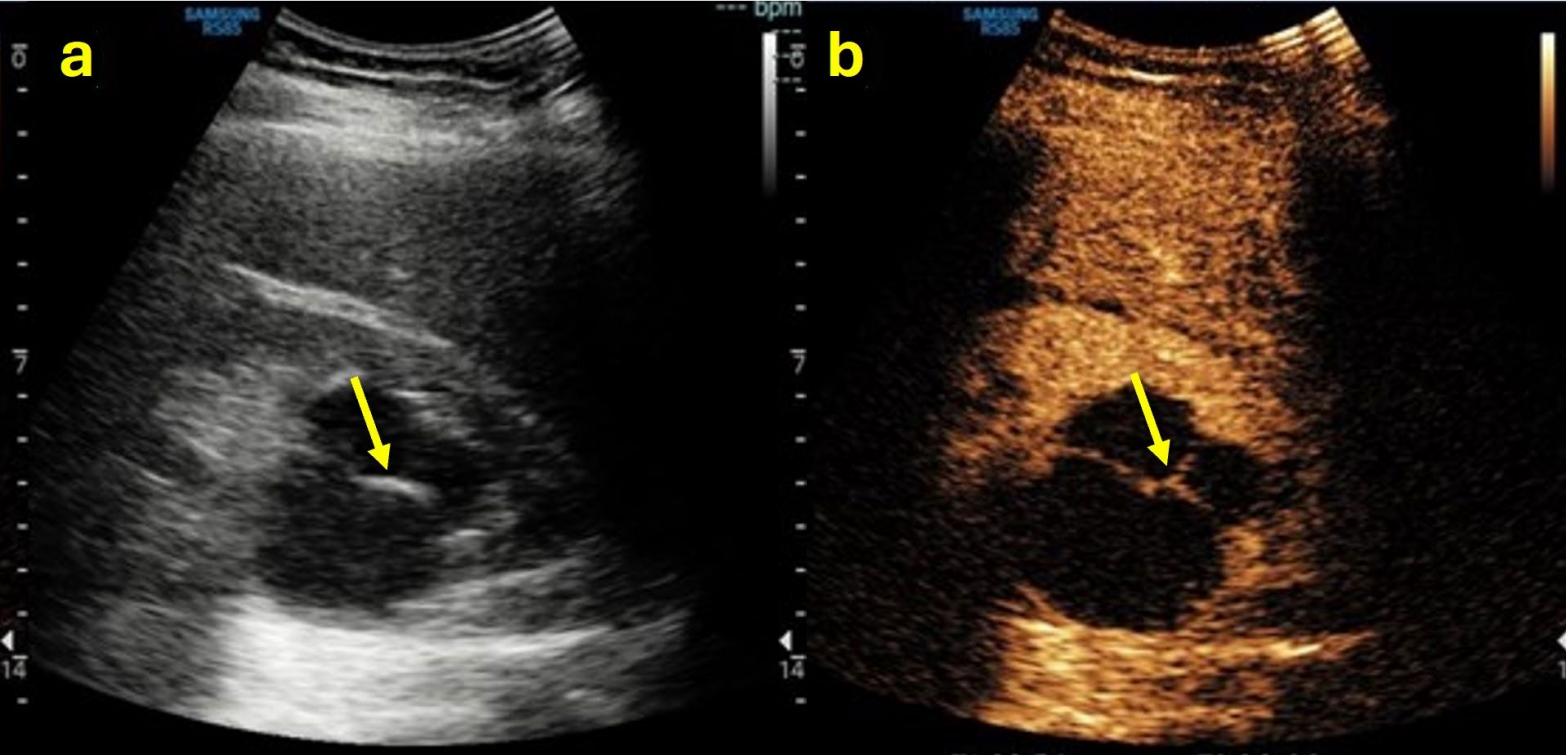
**(a**, **b)** B-mode US image **a** showed a renal cystic lesion presenting septa (yellow arrow); CEUS image **b** showed no contrast enhancement of the wall and septa, more easily detectable, which were thin and not more than three in number. The lesion was classified as Bosniak II

Once it has been ruled out that the lesion is a simple cyst belonging to class I, all features must be carefully assessed to determine the class of the complex cyst. In the case of a cystic lesion that shows the characteristics of belonging to Bosniak class II, the lesion is a benign and minimally complex cyst. In this case, completing the examination with CEUS is not necessary (Table 2). Therefore, B-mode evaluation is sufficient since it characterizes this kind of cyst, except in cases with calcifications, which do not allow evaluation of the content. Calcification is not a sign of malignancy provided that there is no associated suspicious lesion. If CEUS is carried out anyway, it would show no enhancement of the wall and septa, an echogenic content without enhancement, and individual microbubbles running within the tiny vessels in the wall and septa (Fig. 5). However, in the case of a cystic lesion with mixed or echogenic content, it is necessary to perform CEUS to be able to definitively exclude the presence of vegetation.

**Table 2:**
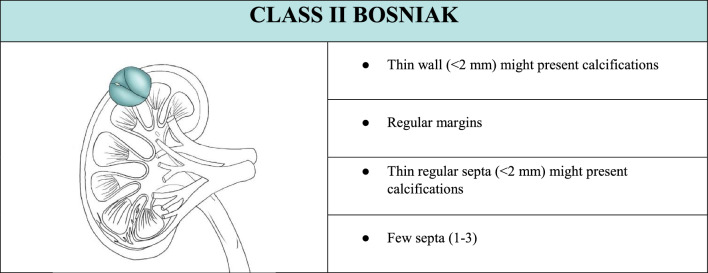
Summary of ultrasound features to define a class II Bosniak cyst, associated with explanatory iconographic image

**Fig. 6:**
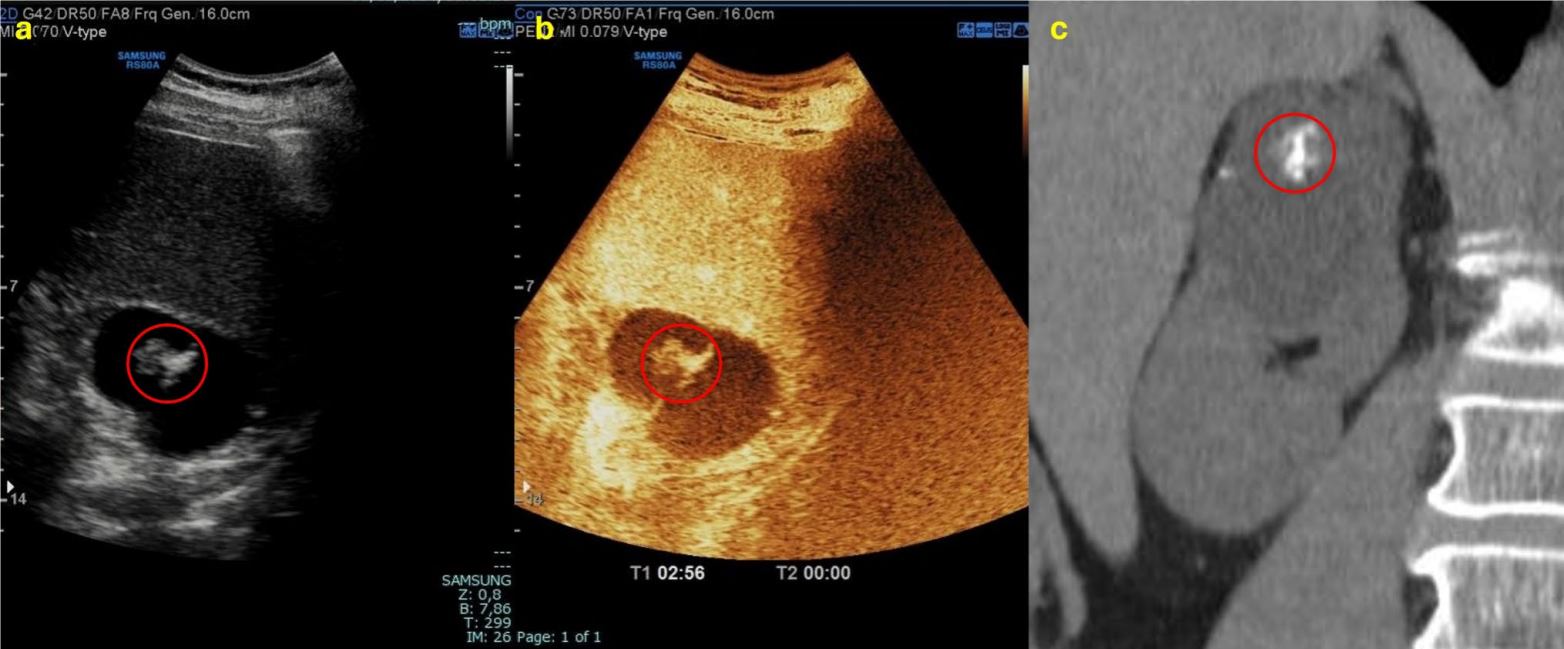
**(a**, **b**, **c)** B-mode US image **a** showed a cystic mass in the right upper pole of the kidney with hyperechoic content due to contextual calcifications (red circle); because of the calcifications, CEUS image **b** did not allow adequate evaluation of eventual enhancing septa; coronal non-contrast CT scan **c** confirmed the presence of calcifications but was not able to better characterize the mass. The mass was classified as Bosniak IIF and required further evaluation with MRI

In the case of a cystic lesion that shows the characteristics of belonging to Bosniak class IIF, the lesion is likely benign and requires imaging surveillance. In the case of a class IIF according to Bosniak, completing the examination with CEUS is necessary. CEUS would show a thin or minimally thickened wall and septa with enhancement, as well as small irregularities. At the same time, intrarenal cysts where differentiation between non-enhancing and enhancing margins cannot be determined are categorized here (Table 3). Additionally, class II cystic lesions that cannot be fully assessed due to the presence of contextual calcifications fall into this class. However, when it is fully impossible to assess the lesion because the calcifications are responsible for artifacts on US and CEUS or do not allow assessment of the wall, content, septa and enhancement on CT, it is advisable to perform contrast-enhanced MRI as a diagnostic examination to assign the correct Bosniak class [20] (Fig. 6).

**Table 3:**
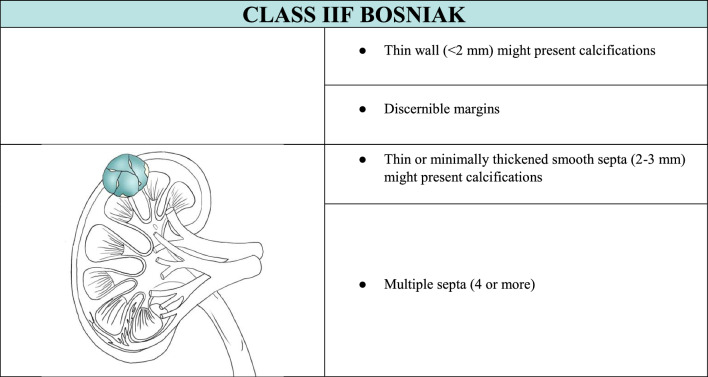
Summary of ultrasound features to define a class IIF Bosniak cystic mass, associated with explanatory iconographic image

**Fig. 7:**
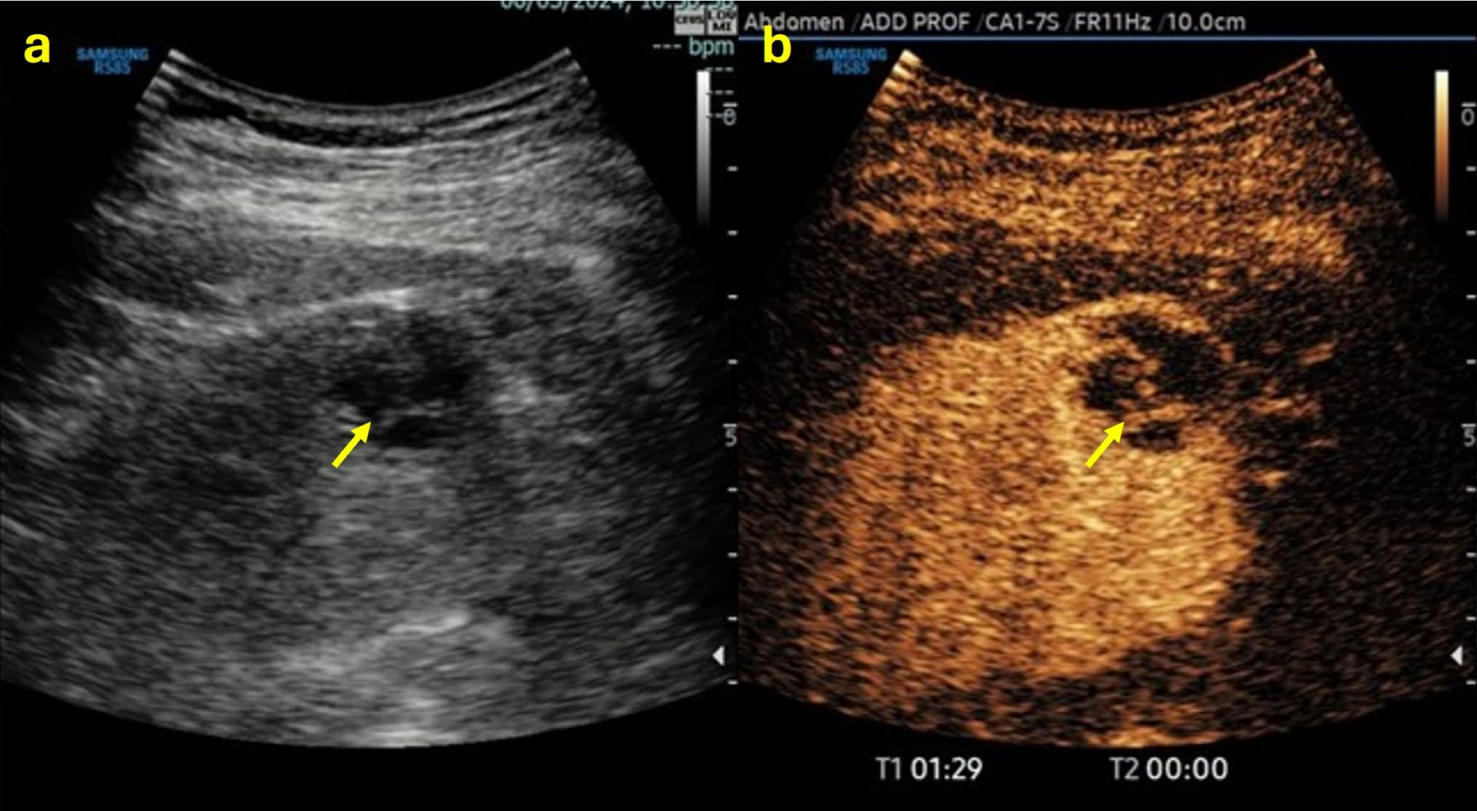
**(a**, **b)** B-mode US image **a** showed a renal anechoic lesion with irregular margins and thickened septa (yellow arrow); CEUS image **b** showed enhancement of the wall and thickened irregular septa (> 3 mm) but excluded the presence of contextual nodules. The mass was classified as Bosniak III

In the case of a cystic lesion that shows the characteristics of belonging to Bosniak class III, the lesion is of uncertain nature. This kind of lesion poses a particular interpretative challenge for the observer because the imaging boundary between benign and malignant lesions is often unclear. In this case, completing the examination with CEUS is necessary (Table 4). CEUS would show enhancing smooth thick walls or septa, and/or enhancing irregular walls (≥ 4 mm) and/or septa (> 3 mm) (Fig. 7).

**Table 4:**
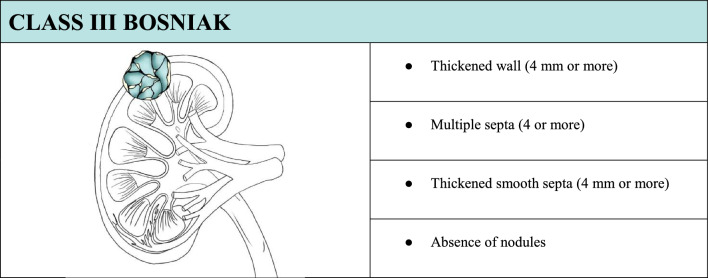
Summary of ultrasound features to define a class III Bosniak cyst, associated with explanatory iconographic image.

**Fig. 8:**
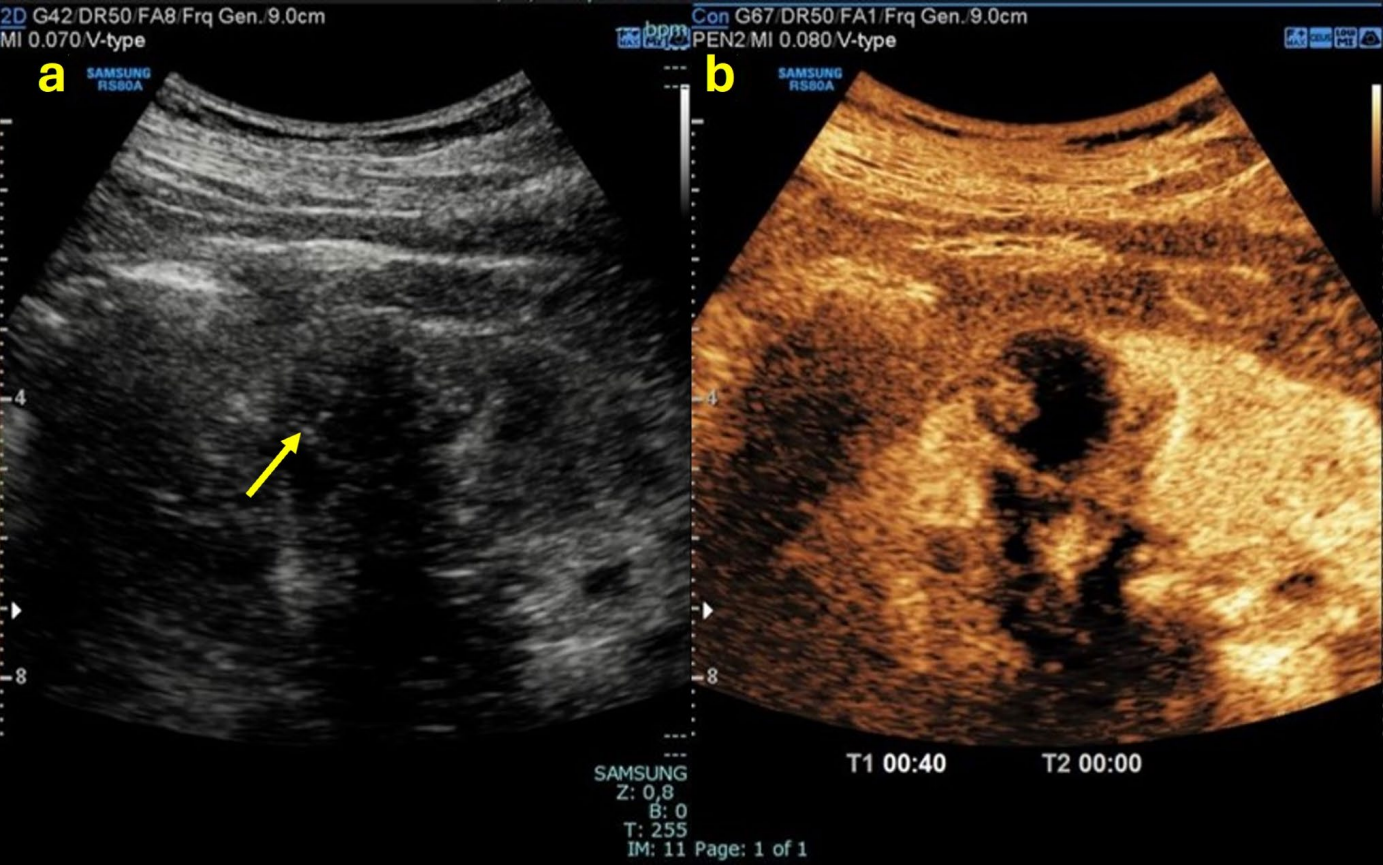
**(a**, **b)** B-mode US image **a** showed an ill-defined hypoechoic renal mass (yellow arrow); CEUS image **b** allowed a better characterization since it showed the anechoic content of the renal mass presenting endoluminal protrusion, and a thickened contrast-enhanced wall. The mass was classified as Bosniak IV

In the case of a cystic lesion that shows the characteristics of belonging to Bosniak class IV, the lesion is likely a malignant cystic tumor. In this case, the lesion presents additional features beyond those of the previous class III. In this case, completing the examination with CEUS is necessary (Table 5). CEUS would show an enhancing smooth thick (> 3 mm) wall or septa and/or enhancing soft-tissue protrusions (Fig. 8).

**Table 5:**
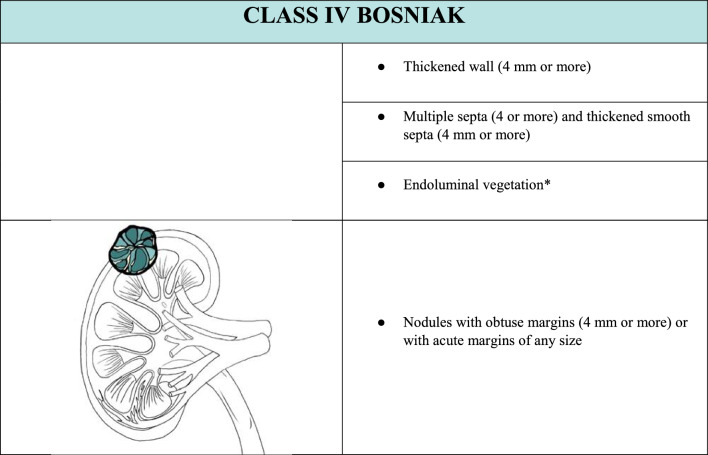
Summary of ultrasound features to define a class IV Bosniak cyst, associated with explanatory iconographic image. *The presence of endoluminal vegetation is a necessary and sufficient condition for classifying the cyst as BIV, even in the absence of the other characteristics listed.

### Contrast-enhanced ultrasound and contrast-enhanced computed tomography

The CECT-based Bosniak cyst classification system has been used for categorizing cystic renal lesions on CEUS, yielding comparable results. However, CEUS tends to upgrade complex renal cystic lesions. Different imaging methods assess various aspects of renal cystic lesions with varying degrees of sensitivity and specificity. Therefore, when assigning the Bosniak category based on CECT and CEUS, it is crucial to consider the differences in imaging techniques to avoid misclassification. Criteria for assessing Bosniak categories based on US and CEUS notably differ from those based on CT and CECT.

CECT measures enhancement using the region of interest (ROI), while CEUS can only determine the presence of enhancement, albeit with higher sensitivity due to its ability to identify single microbubbles within tiny vessels. CECT allows defining a cystic tumor when it presents less than 25% enhancing tissue [21]. In contrast, CEUS excels in detecting septa and precisely evaluating their thickness and irregularities. It is noteworthy that CEUS is highly sensitive in revealing even tiny capillaries, and that thin septa can appear thicker with heavy enhancement if an excessive dose of UCA is injected (microbubble piling and blooming artifact), potentially leading to false upgrades when applying the original Bosniak criteria.

Attenuation is a specific criterion for CECT, and the presence of echogenic content on CEUS could be the corresponding finding for high attenuation, but it is not equivalent since hyperdense cysts can show anechoic content on B-mode US.

Regarding the presence of calcifications, US and CEUS are not appropriate for assessing the lesion due to related artifacts; on the other hand, that may hamper the visualization of any deeper enhancing nodules or septa, making lesion categorization ineffective. CT has a high sensitivity and specificity in detecting the presence of calcifications on the basal scan and with the bone window, but MRI with contrast is required to avoid calcifications impeding an accurate assessment of the enhancement. Therefore, if the calcifications do not allow Bosniak classification, an in-depth diagnostic examination with contrast-enhanced MRI is appropriate since MRI is more sensitive to enhancement than CEUS even in the presence of calcifications [20]. As for the presence of cyst wall calcification with acoustic shadowing, patient habitus or overlying bowel gas might obscure visualization with US and CEUS, limiting the examination result.

Nodules are only seen in Bosniak IV complex renal cysts and are easily distinguished from wall or septal thickening on a CEUS examination.

CEUS reveals a greater complexity in cystic lesions, offering the potential for enhancing lesion characterization and effectively changing therapeutic management. CEUS can improve diagnostic accuracy for cystic renal lesions initially categorized on CECT. Although CECT is the reference standard for Bosniak categories of renal cystic malignancy risk, CECT is inherently inaccurate, with a reported sensitivity of 89.6% and specificity of 65.1% in distinguishing between benign and malignant renal cysts.

**Fig. 9:**
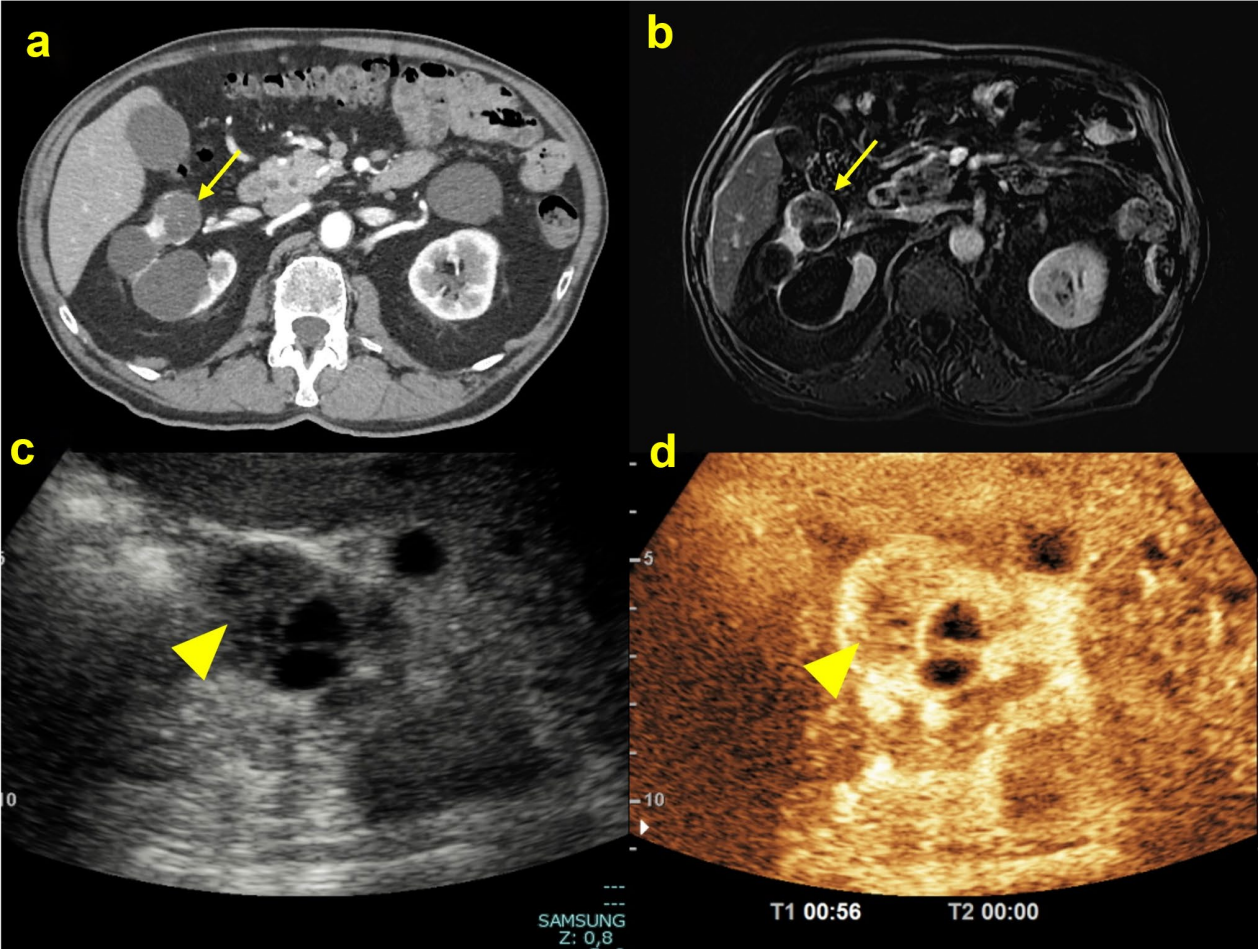
**(a, b, c, d)** Axial CT scan in the arterial phase **A** showed an inhomogeneous renal cystic lesion (yellow arrow) due to the prevalent cystic component; post-contrast T1-p fat-suppressed sequence in the arterial phase **B** confirmed the larger liquid component of the renal lesion (yellow arrow); B-mode ultrasound scan **C** showed a renal lesion with mixed echotexture (yellow arrowhead), with a contextual liquid component; CEUS **D** showed a solid nodule, with > 25% greater contrast gain. The histological diagnosis was clear cell carcinoma

CEUS demonstrates previously undetected features. Minimal septal enhancement is not indicative of malignancy, and an increased sensitivity of CEUS demonstrating enhancing nodules not seen with CECT has been noted. Both upgrading and downgrading of Bosniak categories with CEUS compared to CT imaging are apparent in > 20% of cases (Fig. 9). There is a potential for CEUS to overestimate the Bosniak category, with the ‘real-time’ examination able to demonstrate minor enhancement (a marker of malignant potential). The current view suggests that this is an advantage, rather than a drawback. This requires, however, a fundamental change in imaging assessment of renal cysts, centered on CEUS demonstration of lesion vascularity. When CEUS is inconclusive due to poor visualization, CECT usually permits better characterization and allows the staging of a malignant renal lesion [8].

The presence of enhancement, indicating neovascularization, is the most important factor in determining the need for surgery in cystic renal lesions. Improved CT resolution when compared to the original CECT categories has resulted in fewer indeterminate cyst findings and increased specificity. With CEUS, 31% of renal cysts were attributed to a higher Bosniak category compared to CECT.

In conclusion, focusing on the role of CEUS, the Bosniak classification works well, but it is intrinsically subjective compared to other imaging modalities and is dependent on the observer’s experience. This leads to an unavoidable high degree of inter-observer disagreement [8].

Despite the clinical evidence, the use of CEUS in renal pathology is still off-label, but the ESFUMB has applied very specific guidelines for the use of CEUS in the areas of urology and nephrology, particularly in the study of renal cysts. The American College of Radiology (ACR) cites CEUS as a method with equal appropriateness to CECT and MRI. The EAU guidelines mention CEUS with high sensitivity (100%) and specificity (97%), with a negative predictive value of 100% (κ = 0.95), for the diagnosis of complex renal cysts (Bosniak IIF–III) [16].

### Risk of malignancy and management

The final class of assignment will be related to a percentage of malignancies that defines the management. The first two classes have zero risk of malignancy, but in the case of class IIF, follow-up becomes necessary, and for higher classes, urology consultation is required [21, 22] (Table 6).

**Table 6:** Summary of Bosniak classification describing the type of cysts, the associated risk of malignancy, and its management

Bosniak class	Type of lesion	Risk of malignancy	Management
I	Benign simple cyst	0%	No follow-up
II	Benign minimally complex cyst	0%	No follow-up
IIF	Likely benign cystic mass	5–10%	Follow-up for 5 years: ▪ Every 6 months for 2 years ▪ Once a year for 3 years
III	Uncertain nature	50%	Urology consult
IV	Malignant cystic nature	90%	Urology consult

Identifying the content of a renal lesion (solid or cystic) plays a key role in assignment of the Bosniak class and consequently in the management of the patient, in terms of possible treatment/imaging choice for follow-up. The progression of a lesion towards malignancy is assessed by considering the appearance or increase of solid portions; increase in the number, thickness, or irregularity of septa; and increase in wall thickness. Size growth and growth rate [growth rate (follow-up size minus initial size)/years between measurements] have not been found to correlate with progression as they are often a consequence of fluid accumulation.

Follow-up in class IIF is recommended since about 5–10% of such masses show progression on imaging and those with progression have a probability of about 85% of being malignant. The follow-up can be carried out both with CECT and CEUS imaging since the current evidence shows similar performance for these modalities without a difference in progression to malignancy on follow-up CECT imaging compared to CEUS. Nevertheless, a CEUS examination is suited for follow-up of nonsurgical lesions to detect any morphologic changes such as thickening of septa, appearance of a solid nodule, or contrast-enhanced alterations indicative of progression of the disease when correct imaging acquisition can be guaranteed. In the case of a deep lesion, poor kidney visualization, shadowing from bowel gas or ribs, a non-compliant patient, wall calcification, or a smaller lesion localized within the renal parenchyma, CEUS will not be able to detect and fully characterize the lesion. Therefore, there is the risk that these lesions might be masked during a CEUS examination due to the prominent vascularity of the renal cortex, with the possibility of a lower dose of UCA being helpful. With these issues, further CT or MR imaging is necessary [8].

Najafi et al. demonstrated in their retrospective study that the CEUS examination performed during renal lesion follow-up was consistent with the Bosniak classification in 96% of cases, while only 4% were falsely assessed as malignant [23].

In the case of a class IV lesion, a urologist might consider resection or ablation, while a class III lesion might be suitable for follow-up in patients without comorbidities or with a limited life expectancy [22].

## Conclusion

CEUS, like US, is an operator-dependent method. This approach requires further investigation in cases where the lesion is not visible. However, it is important to consider that, despite its limitations, CEUS results in a higher classification in 31% of renal cysts compared to CT, revealing previously unidentified characteristics. CT has a more established history as it has been in use from 1986 up to the present day, but its main limitations are the use of ionizing radiation and low sensitivity in detecting renal cystic lesion enhancement. In contrast, experience with US is increasing with numerous studies and EFSUMB guidelines since 2020. It is important to not forget that the Bosniak classification has as a primary goal of being a malignancy prediction system, not a comprehensive management algorithm. Patient factors, such as age, comorbidities, life expectancy, preferences, and risk tolerance, must always be considered in a treatment plan and may not reflect the recommendations for each Bosniak class.
